# Digital Measurement of Subjective Experiences in Alzheimer Disease and Related Dementias (AD/ADRD)

**DOI:** 10.2196/71920

**Published:** 2025-11-18

**Authors:** Colin Depp, Jason Holden, Eric Granholm

**Affiliations:** 1 Department of Psychiatry, University of California, San Diego, 9500 Gilman Drive, MC 0737, La Jolla, CA, 92093, United States, 1 8585342542

**Keywords:** Alzheimer's disease, measurement, passive sensing, subjective experience, mobile assessment

## Abstract

Symptoms such as loss of pleasure, agitation, and sadness are subjective experiences that contribute significantly to caregiver burden and health care costs in Alzheimer disease and related dementias (AD/ADRD). However, traditional self-report measures of subjective experiences are limited in AD/ADRD due to cognitive impairments and awareness. Passive sensing, which collects data without active participant input, has emerged as a promising approach to quantify aspects of subjective experiences. Smartphones, wearables, and in-home sensors can quantify mobility, physiology, speech, and social interaction markers of constructs relevant to AD/ADRD. Available research indicates potential but is largely at the proof-of-concept stage. In this Commentary, we discuss several roadblocks to future translation of passive sensing in measuring subjective experiences in AD/ADRD, including technical implementation, data harmonization, validation, ethical and privacy principles. Addressing these challenges could lead to transformative applications to care for AD/ADRD, enabling precise monitoring of behavioral symptoms and related treatment targets, ultimately improving quality of life for persons with AD/ADRD and their caregivers.

## Introduction

Subjective quality of life is a primary concern of persons living with Alzheimer disease and Alzheimer disease-related dementias (AD/ADRD) and their caregivers. Subjective experiences, or an individual’s internal and personal perception of events, emotions, or sensations, include important outcomes in AD/ADRD such as well-being, affect, pain, and loneliness. Additional subjective experiences, like loss of pleasure, agitation, and sadness, are among the behavioral symptoms of dementia that lead to the greatest caregiver burden and health care costs. Reliable and valid measurement approaches are thus critical to quantifying these subjective experiences as outcomes in treatment studies. AD/ADRD produce substantial roadblocks to the measurement of subjective experiences, such as cultural differences in how symptoms are expressed, variability in caregiver interpretation of the experiences of patients, or practical usability challenges associated with sensors in older adults. Self-report measures of subjective experiences can make substantial demands for cognitive abilities impaired in AD/ADRD (memory, verbal fluency, comprehension) and can be biased by cultural milieu, social situation, and memories and schemas of the subjective experience in question. In recent years, technological approaches to gathering data about subjective experiences have been developed, implemented, and tested. Passive sensing can be used to gather data proximal to a variety of experiences that would be difficult or impossible to gather without self-report. Moreover, a promise of passive sensors and related devices is their capacity to gather continuous information that could be used to understand within-person dynamics of subjective experiences, and ultimately better personalize interventions to unique timings and contexts. While there is much enthusiasm about the potential for such tools to support subjective measurement, there remain a number of challenges (and opportunities for innovation) to generate translatable and useful passive measurement tools in AD/ADRD. We briefly summarize some of these challenges and provide accompanying suggestions for future innovators in this area.

## Passive Sensing Methods

 Passive sensing occurs when data are acquired by a device without intentional input from the respondent. Sensing typically uses a variety of devices that can be broadly grouped into three clusters: a) smartphones, b) wearables, and c) in-home sensing devices [1,2]. Common smartphone sensor data streams evaluated in studies of subjective experiences include GPS, accelerometers, Bluetooth, and WiFi data streams. These tools can enable quantification of movement and nearby cell phones, eye movements and facial expressions, and ambient noise and speech features [3]. In addition to sensors, smartphones produce metadata such as calls, texts, app use, and other device interaction data [4]. Also available on mobile devices are more granular device interaction data that has been used to infer cognitive and emotional processes, such as keyboard analytics like errors or typing speed [5]. Wearable sensors like smartwatches and rings produce data on mobility as well as other physiological parameters, such as heart rate variability, sleep patterns, galvanic skin response, temperature, or respiration rate. In-home sensors have a variety of form factors to include ones that are intended to be “invisible” and interactive ones like robots [6]. In-home sensors include infrared or pressure sensors designed to monitor mobility patterns or other interactions within physical spaces. Audio sensors have been used to gather socially-relevant data such as the quantification of linguistic or acoustic speech markers [7]. Video data includes collection from 3D cameras that can be used to generate facial or skeletal models that quantify deviations from normative trajectories [8]. Increasingly, researchers have attempted to integrate multiple sensor streams via computational approaches [9], and create feedback systems for caregivers leveraging passive sensing data [10]. As consumer-grade devices accumulate greater processing power and more novel and powerful sensors are created, it is easy to see why passive sensing research has burgeoned in recent years.

## Challenges in Validating Passive Sensing of Subjective Experiences in ADRD

A first principle in considering and reporting on sensor-based subjective experience research is that subjective experiences are inferred rather than measured directly, as is the case with self-report (see [11] for an excellent summary). Therefore, terminology used in reports should be consistent with the notion that passive sensing is a proxy for but not a direct measure of the construct of interest. This does not mean that sensors cannot be useful for research on subjective constructs. A related point is that the current “gold standard” for validation of passive sensing measures of subjective experience is self-report, which is not free of biases or the impact of cognitive impairments in AD/ADRD. Patients may not recall recent experiences or fully comprehend language and questions. For example, global well-being (*what is your overall well-being?*) can diverge from aggregated immediate experience (*what is your well-being right now?*) measured via ecological momentary assessment (see [12]). It also should not be forgotten that self-report measures can be adapted to people with cognitive impairment or language production difficulties (eg, with visual analog scales [13]), and recent work indicates that active smartphone-based assessments and ecological momentary assessment are feasible and valid in older adults with mild cognitive impairment [14]. Finally, caregiver reports and clinician ratings of patient subjective experiences can also be used as “gold standards” to validate passive sensing measures, but these measures place an added burden on caregivers and providers and are not free of biases or the impact of the observer’s capacity to infer patient experiences.

Central to validation of passive sensing measures in AD/ADRD research is this question: What if “gold standard” subjective experience measurement (eg, a questionnaire) is difficult or impossible to obtain due to the effects of cognitive impairment? As Kourtis et al [15] point out that in late-life depression and Alzheimer’s disease, multiple passive sensors show promise in detecting multiple subjective experiences, like mood, loneliness, suicide risk, agitation, daily life functioning, and dementia onset and progress, but work is only beginning with these measures. For example, Au-Yeung et al [16] evaluated a home-based mobility sensor in adults with a dementia diagnosis, and documented mobility patterns in an effort to examine within-person dynamics of agitation and apathy. A pilot study by Galambos et al [17] tested an early dementia detection model in older adults, with home-based sensors detecting changes in the amount of time spent in the bedroom, in the living room, and mealtime activities that have congruence with health assessments. Other passive sensing methods use infrared sensors to assess time spent in the house versus out of the house [18] as well as the speed of ambulation through the house [19], which have correlated with cognitive function changes. Another study [20] found that wireless home-based sensors captured differences in activities of daily living patterns between dementia and healthy individuals. Smartphone-based Bluetooth detection of nearby cell phones, actigraphy, and GPS data have been used for predicting loneliness in college students [21] and between Bluetooth features and depression [3], but this work is only beginning in AD/ADRD.

As several reviews of the literature of sensor-based systems of subjective experiences have concluded [1,22] the great majority of research studies to date have been at the “proof of concept” phase. Few studies have been replicated, and few technologies have been evaluated in samples designed for rigorous validation. For research on passive sensing to advance the replicability of sensing, it could be useful to adapt systematic and coordinated methods being used to improve self-report. An enormous amount of effort has gone into developing researcher toolkits like PROMIS (Patient-Reported Outcomes Measurement Information System) and NeuroQoL (Quality of Life in Neurological Disorders) [23]. These National Institutes of Health–sponsored projects spearheaded development of common measures of subjective constructs by rigorous item development, reliability and replicability testing in diverse samples so as to mitigate bias, and application of advanced psychometric techniques toward item selection, short-form development to maximize scalability, along with establishment of convergent and divergent validity specifically in people with diminished cognitive ability (eg, use of visual analog scales). Perhaps some processes involved in these coordinated efforts could serve as models to enhance the utility of passive sensing.

 Key roadblocks to creating such a uniform approach to enhancing the replicability of passive sensor data are the intricacies of feature extraction and computational processing of data streams. To move passive sensing toward a more standardized and sharable method, there are open science solutions to enhance transparency, including platforms to harmonize data standards, for example, the Collaborative Aging Research using Technology Initiative (CART) [24]. Repositories for research protocols and other collaborative approaches, such as checklists [25], are available to support best practices in data collection, processing, and validation. Application of Findable, Accessible, Interoperable, and Reusable (FAIR) [26] principles to data collection processes would enable the needed comparison of the impact of different devices and software versions on outputs. These initiatives should support greater aggregation of results across studies to address fundamental questions about sensing relevant subjective experiences that persist: Which sensor or combination of sensors is most accurate and reliable in sensing aspects of which subjective experiences? How long and at what data density is required to obtain valid results? Which sensors offer the greatest balance of practicality in implementation, unobtrusiveness, cost and data processing demands, and validity and reliability? What are the best practices specific to people with ADRD and their caregivers in maximizing adherence?

 While the coming years will undoubtedly bring a plurality of new, more-sensitive sensors, and new approaches to measuring aspects of subjective experience applicable to ADRD, it is essential that this research accompany robust consideration of the principles of the Belmont report in respect to respect for persons, beneficence, and justice. For the same reasons that self-report is hampered, so is informed consent in ADRD. Tools to simplify informed consent and assess and improve decisional capacity are available [24,27], but the technical and privacy considerations surrounding passive sensing (eg, comprehension) can present marked challenges even for people without cognitive impairment. Resources such as guidelines to identify best practices in obtaining proxy or individual informed consent and granular choices surrounding the type of data collected could maximize respect for the individual in the informed consent setting [28]. The industry has a major role in this, including the deployment of health research infrastructure and gatekeeping functions to ensure privacy standards are met and maintained during the research, alongside software updates.

 Another piece of the puzzle is engagement in the community in regard to the setting of the sensor and also the results of the research. Few sensors or devices used in the collection of data in sensor studies were designed with older adults in mind. For example, wearable sensors may not be calibrated to age-associated factors such as the thinning of skin, and conclusions from those sensors may be erroneous [29]. Given that caregivers are often essential partners in the deployment and sustainment of data collection, their unique needs and challenges need to be considered [30]. Therefore, part of the researcher’s task is to partner with older adults, caregivers, and other stakeholders through user-centered design before deployment [31]. As part of this process, researchers should query participants’ desires for the return of information on sensing data, including at the individual level [32]. Community-engaged research approaches may be one way to expand diversity of inclusion in sensor-based research, which will be essential to understand biases that may be present.

## Conclusion 

Paradoxically, these many challenges to passive sensing in AD/ADRD stem from the very same reasons why such approaches offer promise, pressing unmet needs. The behavioral symptoms of dementia, including agitation or loss of pleasure, are among the single largest drivers of caregiver distress and institutionalization. Making the case for passive sensing in behavioral symptoms even more compelling are the potential risks, costs, and harms of current treatments for behavioral symptoms (eg, side effects, institutionalization). There are emerging systems that infer these subjective symptoms by use of passive sensing alongside contemporaneous dynamic data from potential contributors to the onset and sustainment of these symptoms that could be targets for intervention. Clinical translation opportunities could include more precise monitoring of change in subjective experiences that are targeted by interventions (eg, pharmacologic or nonpharmacologic treatments targeting loss of pleasure). Given the continuous nature of passive sensing, idiographic approaches are possible, wherein personalized interventions could be developed through user-centered design [33] in a just-in-time fashion based on data garnered from the individual. We note that similar to more active digital health solutions, validation is only one step and clinical implementation accompanies many additional challenges (eg, cost, urgent responses, provider burden) [34]. Thus, coordinated efforts at addressing roadblocks to the translation of passive sensing of subjective experience in ADRD could lead to transformative approaches that address critical unmet needs.

## Abbreviations

AD/ADRD: Alzheimer disease and related dementias
